# A Mosaic PIK3CA Mutation in a Moroccan Female: Exploring the Diagnostic Challenges of PIK3CA-Related Overgrowth Spectrum

**DOI:** 10.7759/cureus.36996

**Published:** 2023-04-01

**Authors:** Mohamed Ahakoud, Hanae Daha Belghiti, Hajar Ihlal, Laila Bouguenouch

**Affiliations:** 1 Medical Genetics and Oncogenetics Laboratory, Hassan II University Hospital, Fez, MAR

**Keywords:** pik3ca, pi3k/akt/mtor signaling pathway, sanger sequencing, pik3ca gene, genetic mosaicism, pik3ca-related overgrowth spectrum

## Abstract

The PIK3CA-related overgrowth spectrum (PROS) encompasses a group of rare disorders characterized by the overgrowth of various body parts, driven by mutations in the PIK3CA gene. This study presents a case of a Moroccan female patient with PROS, demonstrating a phenotype associated with genetic mosaicism in the PIK3CA gene. A multidisciplinary approach, involving clinical examination, radiological assessment, and genetic and bioinformatic analyses, was employed for diagnosis and management. Next-generation sequencing and Sanger sequencing identified a rare variant, c.353G>A, in exon 3 of the PIK3CA gene, not detected in leukocyte DNA but confirmed in tissue biopsy samples. The comprehensive analysis of this case furthers our understanding of PROS and highlights the importance of a multidisciplinary approach to the diagnosis and management of this rare disorder.

## Introduction

The PIK3CA-related overgrowth spectrum (PROS) is a group of rare disorders characterized by the overgrowth of various body parts, including bones, skin, and blood vessels. The disorders are caused by mutations in the PIK3CA gene, which encodes the p110α catalytic subunit of the phosphoinositide 3-kinase (PI3K) enzyme. This gene is essential for regulating cell growth, proliferation, and survival, and mutations in PIK3CA can lead to abnormal activation of the PI3K/AKT/mTOR signaling pathway [1]. PROS affects many different systems in the body and can cause a wide range of symptoms, including skin overgrowth, bone deformities, and cardiovascular abnormalities [2].

Several studies have reported the clinical and genetic characteristics of PROS in different populations [3,4]. A molecular diagnosis of PROS can be challenging due to the variability of the phenotype and the lack of specificity of the clinical features [3]. Therefore, a multidisciplinary approach is needed for the diagnosis and management of PROS, involving geneticists, pediatricians, dermatologists, and other specialists [5].

The treatment of PROS involves the use of low-dose sirolimus [6] and alpelisib [7]. A systematic review found promising results for the efficacy of alpelisib in treating head and neck lymphatic malformations [8]. However, further research is required to fully assess the safety and efficacy of these treatments for PROS. The PI3K/AKT/mTOR pathway is believed to play a critical role in the development of PROS [1]. Targeted therapy is being explored as a potential treatment option for PROS [9].

In this paper, we report a Moroccan female case of PROS presenting a phenotype associated with genetic mosaicism in the PIK3CA gene. This mosaicism was identified by DNA analysis of tissue biopsy samples but was not present in lymphocyte DNA.

## Case presentation

**Figure 1:**
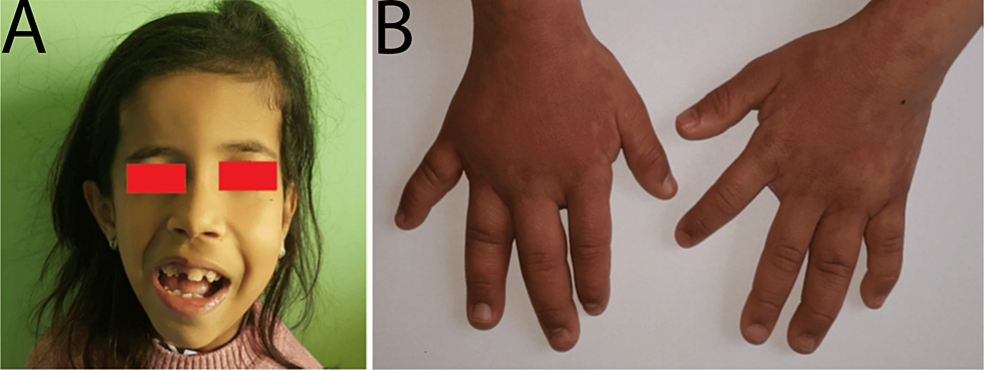
Clinical manifestations: (A) left hemifacial hypertrophy and (B) livedo-like lesions.

The patient was a seven-year-old female born with a birth weight of 4.400 kg, length of 54 cm, and head circumference of 41 cm. She was diagnosed with congenital hip dislocation in the neonatal period. The patient showed developmental delay, with sitting achieved at seven months and no standing or walking. She also had a speech delay. Physical examination revealed global developmental delay, left-sided hemihypertrophy, skin and joint hyperlaxity, and multiple cutaneous anomalies, such as angiomas on the back and limbs, hamartomas on the neck and left upper limb, livedo-like lesions on the left lower limb, and painful tumefaction of the left forearm. She also had an umbilical hernia (Figure 1).

In 2016, at the age of seven years, she was referred to the medical genetics unit for further evaluation of her overgrowth phenotype and multiple cutaneous anomalies. A detailed physical examination showed that the child was taller and heavier than her peers and had an asymmetric overgrowth of her left arm and leg, with a left upper limb circumference of 25 cm (normal range: 16-20 cm) and left lower limb circumference of 36 cm (normal range: 24-28 cm). She had a limited range of motion of her left elbow and knee and a painful tumefaction of the left forearm that had been present for several months.

Laboratory examinations, including complete blood count and renal function tests, were normal. Radiological examination showed normal renal and abdominal ultrasound, but an ultrasound of the soft tissue (tumefaction of the left forearm) showed a vascular formation likely a thrombosis, and brain magnetic resonance imaging (MRI) revealed left cerebral hemisphere hypertrophy with midline shift to the right, dilatation of the ventricular system, and cortical fissures.

**Figure 2:**
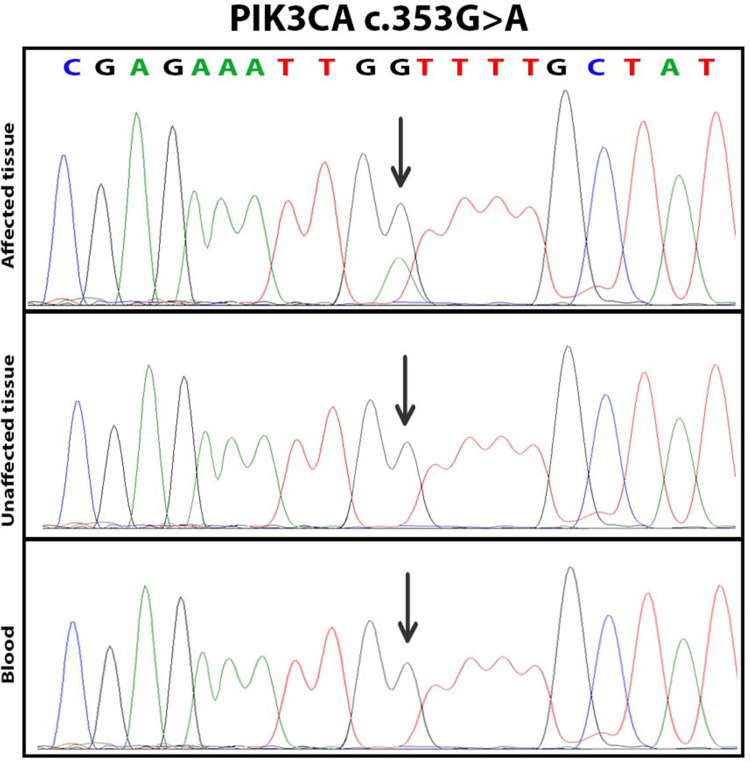
Partial electropherogram of PIK3CA confirming the presence of PIK3CA c.353G>A in the affected tissue sample only.

A genetic analysis was performed using next-generation sequencing and confirmed by Sanger sequencing. The analysis revealed a variant in exon 3, c.353G>A, leading to a p.Gly118Asp change (Figure 2). This variant is rare and not found in population studies (Exome Sequencing Project, Exome Aggregation Consortium) but it has been identified as a somatic variant in several malignant tumors (COSMIC) and in a patient with Cowden disease [10] and angio-osteo-hypertrophic syndrome. Bioinformatic analyses predict that this variant is probably deleterious and it affects a highly conserved amino acid. It has been reported that this variant could weaken the interaction with the PIK3R1 protein and increase the catalytic activity of PIK3CA [11]. This deleterious variant confirms the molecular diagnosis of PROS. The mosaic feature of the variant found in the lesional sample is confirmed by the absence of detection of this variant by Sanger sequencing on leukocyte DNA.

## Discussion

**Figure 3:**
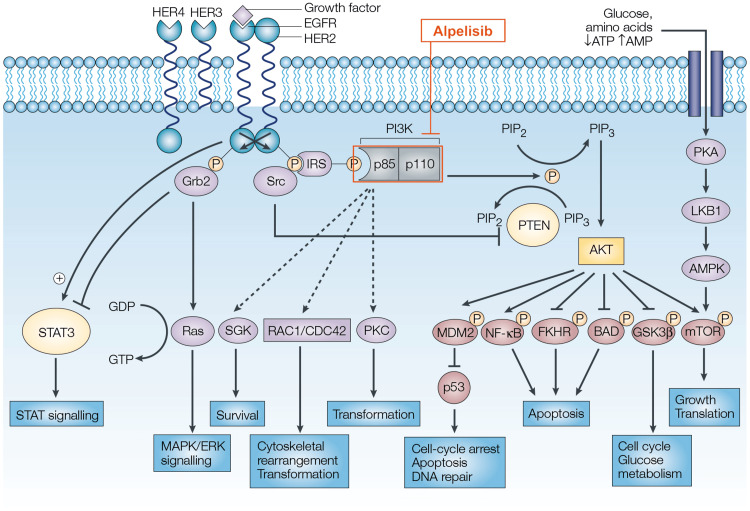
Schematic of signaling through the phosphatidylinositol 3-kinase (PI3K)/AKT pathway. HER2: human epidermal growth factor receptor 2; HER3: human epidermal growth factor receptor 3; HER4: human epidermal growth factor receptor 4; EGFR: epidermal growth factor receptor; AMP: adenosine monophosphate; ATP: adenosine triphosphate; Grb2: growth factor receptor-bound protein 2; IRS: insulin receptor substrate; PI3K: phosphoinositide 3-kinase; PIP2: phosphatidylinositol 4,5-bisphosphate; PIP3: phosphatidylinositol (3,4,5)-trisphosphate; PTEN: phosphatase and tensin homolog; PKA: protein kinase A; PKC: protein kinase C; ERK: extracellular signal-regulated kinase; FKHR: forkhead; GDP: guanosine diphosphate; GSK3: glycogen synthase kinase 3; MAPK: mitogen-activated protein kinase; NF-κB: nuclear factor-κB; PKC: protein kinase C; STAT: signal transducer and activator of transcription; mTOR: mammalian target of rapamycin; LKB1: liver kinase B1; AMPK: AMP-activated protein kinase; MDM2: mouse double minute 2; Cdc42: cell division control protein 42; Rac1: Ras-related C3 botulinum toxin substrate 1; SGK: serum- and glucocorticoid-inducible kinase. Used with permission from [16].

PROS is a group of rare and heterogeneous disorders characterized by somatic mosaic mutations in the PIK3CA gene [12-14]. The PIK3CA gene encodes the p110α catalytic subunit of PI3K, which is a critical component of the PI3K/AKT/mTOR signaling pathway, regulating cell growth, proliferation, and survival [12,15]. Mutations in the PIK3CA gene lead to the overactivation of the PI3K/AKT/mTOR pathway (Figure 3), resulting in aberrant cell growth and the development of various overgrowth phenotypes [1,13-15].

The clinical presentation of PROS is highly variable, ranging from isolated overgrowth to multisystem involvement [8,17,18]. Common features include asymmetric overgrowth, vascular malformations, lipomatous overgrowth, and various cutaneous anomalies [17-19]. In this case, the patient exhibited left-sided hemihypertrophy, multiple cutaneous anomalies, and developmental delay, consistent with the manifestations of PROS.

The diagnosis of PROS is primarily based on clinical findings and confirmed by molecular testing [3,19]. In our patient, the genetic analysis identified a somatic mosaic mutation in the PIK3CA gene (c.353G>A, p.Gly118Asp), confirming the diagnosis of PROS. This variant has been previously reported in patients with Cowden disease and angio-osteo-hypertrophic syndrome and is predicted to be deleterious [10,20]. The mosaic nature of the mutation was confirmed by the absence of detection in leukocyte DNA.

Management of PROS is multidisciplinary and primarily focused on symptom relief and improving the quality of life [1,8,18]. For patients with vascular malformations, medical management with sirolimus has shown promise in reducing lesion size and improving symptoms [6]. In addition, targeted therapies such as alpelisib, a PI3Kα inhibitor, have shown efficacy in treating patients with PROS [7,10]. However, further research is needed to determine the long-term safety and efficacy of these targeted therapies.

## Conclusions

In conclusion, this case study of a Moroccan female with a mosaic PIK3CA mutation illuminates the complex clinical manifestations associated with PROS and the critical role played by molecular testing, such as next-generation sequencing, in achieving a definitive diagnosis. By employing a multidisciplinary approach, involving specialists in genetics, radiology, and pediatrics, the comprehensive evaluation and management of patients with PROS can be ensured. Additionally, this case emphasizes the potential of targeted therapies, such as PI3K/AKT/mTOR inhibitors, in providing more effective and individualized treatment for patients with PROS. Further research is warranted to explore the molecular mechanisms underlying PROS pathogenesis and to advance the development of novel therapeutic strategies tailored to the specific molecular alterations present in affected individuals.
